# Hepatitis C Cirrhosis, Hepatitis B Superimposed Infection, and the Emergence of an Acute Portal Vein Thrombosis: A Case Report

**DOI:** 10.7759/cureus.39839

**Published:** 2023-06-01

**Authors:** Lauren Kasmikha, Naoshin Khan, Mohamed Ramzi Almajed, Abigail Entz, Syed-Mohammed Jafri

**Affiliations:** 1 Internal Medicine, Wayne State University School of Medicine, Detroit, USA; 2 Internal Medicine, Henry Ford Hospital, Detroit, USA; 3 Gastroenterology and Hepatology, Henry Ford Hospital, Detroit, USA

**Keywords:** portal vein thrombosis (pvt), acute portal vein thrombosis, hcv cirrhosis, superimposed infections, hepatitis c infection, hepatitis b infection, decompensated cirrhosis

## Abstract

Acute portal vein thrombosis (PVT) is a complication of liver cirrhosis. The presence of viral infections such as hepatitis B (HBV) and hepatitis C (HCV) can further increase cirrhotic patients’ risk of developing PVT, especially in the rare case when there is superinfection with both HBV and HCV.

We present a patient with HCV cirrhosis whose clinical condition was decompensated secondary to the development of superimposed HBV infection, who developed acute PVT during hospitalization. This case offers a unique presentation of acute PVT that developed within several days of hospitalization for decompensated liver disease, as proven by the interval absence of portal venous flow on repeat imaging. Despite the workup on the initial presentation being negative for PVT, reconsideration of differentials after the change in our patient’s clinical status led to the diagnosis. Active HBV infection was likely the initial trigger for the patient’s cirrhosis decompensation and presentation; the subsequent coagulopathy and alteration in the portal blood flow triggered the development of an acute PVT.

The risk for both prothrombotic and antithrombotic complications remains high in patients with cirrhosis, a risk that is vastly increased by the presence of superimposedinfections. The diagnosis of thrombotic complications such as PVT can be challenging, thus stressing the importance of repeat imaging in instances where clinical suspicion remains high despite negative imaging. Anticoagulation should be considered for cirrhotic patients with PVT on an individual basis for both prevention and treatment. Prompt diagnosis, early intervention, and close monitoring of patients with PVT are crucial for improving clinical outcomes. The goal of this report is to illustrate diagnostic challenges that accompany the diagnosis of acute PVT in cirrhosis, as well as discuss therapeutic options for optimal management of this condition.

## Introduction

Acute portal vein thrombosis (PVT) is a complication of liver cirrhosis that can lead to increased morbidity and mortality, as evidenced by increased rates of hepatic decompensation and association with the severity of liver disease [1,2]. PVT is defined as the obstruction of the portal vein or the intrahepatic portal vein branches by a thrombus [3,4]. It is a rare and severe complication that presents in patients with cirrhosis but can also present in patients without liver dysfunction. The prevalence of PVT is estimated to be 1% in compensated hepatic cirrhosis, up to 28% in decompensated cirrhosis, and up to 44% in patients with both cirrhosis and hepatocellular carcinoma [5]. According to a meta-analysis performed by Xian et al., patients with both cirrhosis and PVT had lower survival rates at both one and three years than cirrhotic patients without PVT [1].

Risk factors for PVT development include hypercoagulability, endothelial dysfunction, and changes in the flow dynamics of portal blood [6-8]. The presence of viral infections such as hepatitis B (HBV) and hepatitis C (HCV) can further increase the risk of PVT in patients with cirrhosis, especially those with decompensated liver disease, although the mechanism is poorly understood. In rare cases, both HBV and HCV infections can be present in the same patient, which is referred to as a superimposed HBV on HCV infection. The presence of this condition can accelerate the progression of liver decompensation and increase the risk of complications, including PVT.

In this case, we present a patient with decompensated HCV cirrhosis who presented with acute worsening of his condition secondary to superimposed HBV infection and developed an acute PVT during the hospitalization, resulting in further clinical deterioration.

## Case presentation

A 65-year-old man presented with a one-month history of generalized fatigue, jaundice, nausea, and weight loss secondary to poor oral intake. In addition, he reported mild pruritus, dark urine, acholic stools, and easy bruising during this time. He was known to have decompensated liver cirrhosis secondary to HCV; he was Child-Pugh Class B and developed complications including jaundice, ascites, hepatic encephalopathy, and esophageal varices. He received treatment for HCV and achieved a sustained virologic response.

**Figure 1:**
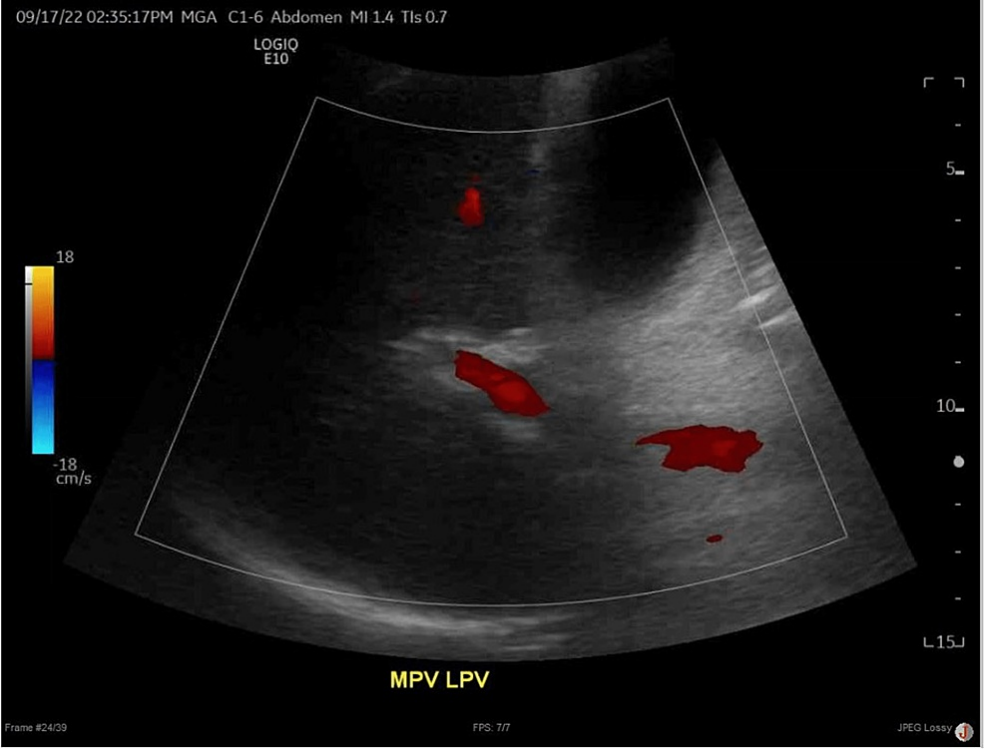
Duplex ultrasonography demonstrating patent hepatic and portal vasculature with appropriate directional flow and duplex waveforms.

Our patient was alert and oriented, and an examination was notable for scleral icterus, abdominal distension, and bilateral leg swelling. Blood workup showed new liver profile abnormalities with aspartate transaminase (AST) of 467 IU/L, alanine transaminase (ALT) of 470 IU/L, and total bilirubin of 10.9 mg/dL. His Model for End-Stage Liver Disease (MELD)-Na score was 26. Hepatitis C RNA was undetectable. Hepatitis B surface antigen and core antibody were positive, surface antibody was negative, and DNA level was 1,481,240 IU/mL, demonstrating a chronic infection state. Of note, he had a positive hepatitis B surface antigen eight years prior, but the result was never followed up with further testing. Ultrasonography with Doppler and CT liver demonstrated patent vasculature (Figure 1) and evidence of cirrhosis with stigmata of portal hypertension but without evidence of ascites or hepatocellular carcinoma. The impression for our patient’s presentation was decompensated liver disease in the setting of active HBV infection, and he was treated with entecavir for antiviral therapy, as well as furosemide and spironolactone for the treatment of ascites and edema. Entecavir was later changed to tenofovir.

**Figure 2:**
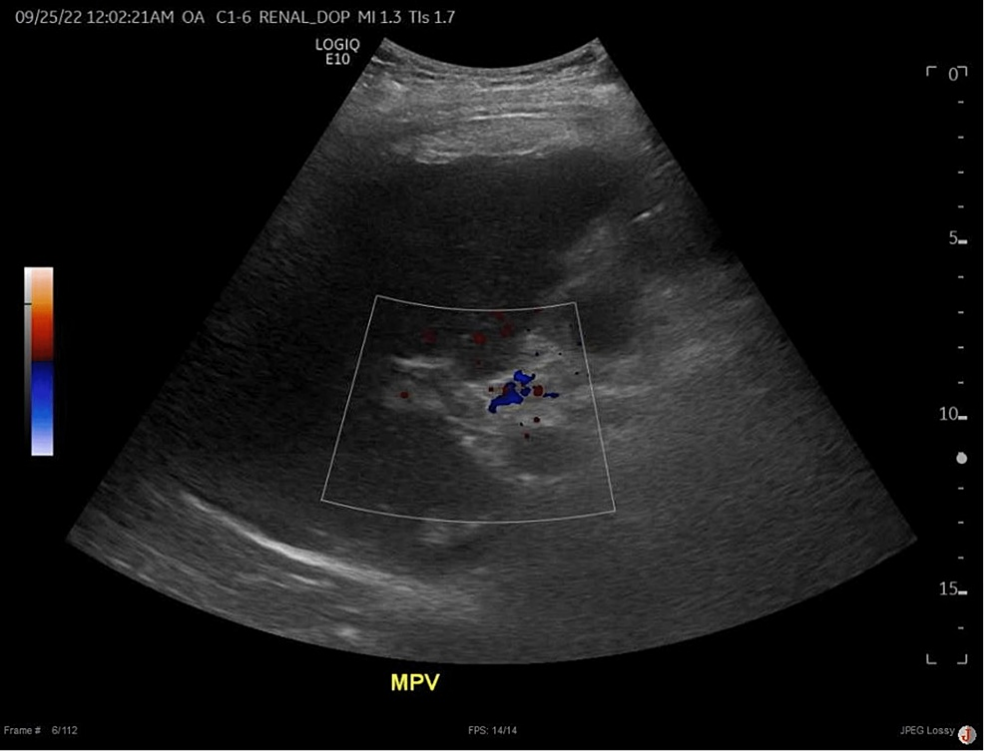
Repeat duplex ultrasonography eight days after the initial examination, revealing absent flow in the portal vasculature suspicious for a portal vein thrombosis. Hepatic vasculature remains patent with unchanged flow.

The patient initially responded to treatment and showed improvement in his symptoms and laboratory abnormalities, as evidenced in Table 1. However, five days after the presentation, he became confused with hepatic encephalopathy, diffuse abdominal pain and tenderness, and increased icterus. He was managed with lactulose and rifaximin for hepatic encephalopathy with minimal improvement. He was initially started on broad-spectrum antibiotics, including vancomycin and piperacillin/tazobactam; however, they were discontinued after the infectious workup was unremarkable. His liver profile worsened with AST of 1,147 IU/L, ALT of 407 IU/L, and total bilirubin of 42.3 mg/dL. The MELD-Na score increased to 39, and a liver transplant workup was initiated. He continued to deteriorate, and his care was escalated to the intensive care unit where a repeat ultrasonography with Doppler demonstrated an interval absence of color flow visualization in the main, right, and left portal veins indicative of acute PVT (Figure 2). Management with intravenous heparin resulted in improvement in laboratory markers. The patient eventually developed acute renal failure, acute respiratory failure, and significantly altered mental status. In the setting of his clinical deterioration, a family meeting took place and the decision was made to transition to comfort care with palliative measures per the patient’s wishes after which he shortly died.

**Table 1:** Liver function and coagulation factors over the course of the patient’s hospital stay, starting on admission, five days after presentation and upon escalation to the ICU, and 10 days after presentation. AST = aspartate transaminase; ALT = alanine transaminase; PT = prothrombin time; PTT = active partial thromboplastin time

	Reference range	Day of admission	Three days after the presentation	Five days after the presentation	Ten days after the presentation
AST	<35 IU/L	467	204	1,147	221
ALT	<52 IU/L	523	378	407	93
Total bilirubin	<1.2 mg/dL	31.1	28.6	42.0	41.0
Direct bilirubin	0.0–0.3 mg/dL	14.9	12.6	24.9	26.0
PT	11.5–14.5 seconds	20.4	21.2	29.3	484.8
PTT	22.0–36.0 seconds	38.0	39.1	47.0	143.0
Platelet count	150 - 450 K/µL	78	93	59	50

## Discussion

Acutely decompensated cirrhosis is the development of cirrhosis-related complications, including ascites, hepatic encephalopathy, variceal bleeding, and bacterial infections [9]. Acute decompensation often progresses to acute-on-chronic liver failure, as well as the development of multiorgan failure [9]. Systemic inflammation, cardiocirculatory dysfunction, and metabolic alterations contribute to the tissue injury that leads to this progression to end-stage liver disease with extrahepatic organ failure [9]. For patients whose clinical status transitions from decompensation into end-stage disease, the MELD-Na is a scoring system used to evaluate potential candidates for liver transplantation but has other applications, including predicting mortality in fulminant hepatic failure, alcoholic hepatitis, cirrhosis with infections, variceal bleeding, trauma, and hepatorenal syndrome, among other complications [10]. The components of the MELD-Na score include serum bilirubin, serum creatinine, international normalized ratio, and serum sodium [10,11]. MELD-Na scores range from six to 40 and higher scores correlate with increased disease severity and priority for organ allocation [12]. The inclusion of hemostatic tests to the MELD-Na scoring system allows for the assessment of hepatocyte function in liver disease and measures the liver’s ability to synthesize clotting factors; this can be used to assess patients’ risk for hematological complications such as PVT in cirrhosis [13].

Various risk factors contribute to the development of PVT in patients with cirrhosis. Major contributing factors have been shown to include male sex, previous treatment for portal hypertension (e.g., sclerotherapy, transcutaneous intrahepatic portosystemic shunt (TIPS), shunt surgery, splenectomy), Child-Pugh Class C, and alcoholic liver disease [6]. Other coexisting conditions include Budd-Chiari syndrome, a hypercoagulable state caused by thrombophilic conditions, and hereditary or acquired prothrombotic factors [6-8]. Patients with cirrhosis have a dynamic fluctuation between a prothrombotic and an antithrombotic state as the severity of liver dysfunction altered the hemostatic balance; it results in reduced production of procoagulants and platelets while also causing decreased levels of anticoagulants such as protein C or antithrombin [14]. Disruption of this balance can predispose patients with cirrhosis to PVT development. A study by Zocco et al. showed that the loss of antithrombotic proteins, and, subsequently, the extent of hemostasis activation, is positively related to the severity of liver cirrhosis, which, in turn, contributes to higher PVT incidence [13].

Patients with more pronounced portal hypertension as a consequence of liver cirrhosis are more likely to be vulnerable to hemodynamic changes in portal vein flow. This manifests as low-flow or reverse-flow states that eventually predispose to thrombosis [6]. An inverse relationship has been identified between portal vein flow velocity and Child-Pugh score as an assessment of cirrhosis severity. Velocity was shown to be lower in patients with more advanced cirrhosis, Class C, than in those with less advanced cirrhosis, Class A or B cirrhosis. Further, stagnation of portal blood flow has been postulated as one of the most prognostic factors for PVT development. This is due to the active thrombin generated in the portal circulation not being washed away by an adequate flow rate [13].

Acute viral hepatitis has also been proposed as a contributor to increased thrombotic risk in cirrhosis patients. By inducing inflammatory changes in the endothelium of the portal vein system, the coagulation cascade can be activated and result in increased PVT risk [14]. In patients with concomitant viral hepatic infections and liver cirrhosis, a multifactorial proinflammatory and prothrombotic state significantly increases the risk of PVT [14]. Various studies have shown that the incidence of decompensated liver disease is higher in patients with HCV cirrhosis and superimposed HBV infection than in those with HCV infection alone. HBV superinfection may also be implicated in the development of cirrhosis decompensation among patients with HCV cirrhosis, often manifesting as fulminant hepatitis. Timely identification of active hepatitis infection and appropriate treatment with antiviral therapy is recommended to prevent recurrent complications, including PVT, as well as to mitigate disease progression [15].

Regardless of etiology, acute PVT can present major diagnostic challenges to clinicians, as its presentation can vary. In patients with cirrhosis, PVT can be asymptomatic and an incidental finding or it can be symptomatic with decompensation; symptoms include the onset or worsening of ascites, bleeding varices, portal hypertensive gastropathy, or intestinal infarction [7,9]. Duplex ultrasonography is the diagnostic modality of choice for PVT, as it is non-invasive, rapidly available, and provides diagnostic accuracy with a negative predictive value of 98% [6,16]. The use of color Doppler with ultrasound allows practitioners to differentiate between the presence of partial or complete occlusion of the portal vein, with the ability to reveal patent luminal areas with flow downstream from the thrombus. However, despite its high sensitivity, color Doppler ultrasound is often difficult to perform satisfactorily, and, thus, cannot always provide confirmation with high certainty [7]. Ultrasound can also be challenging in patients with cirrhosis due to technical limitations in body habitus, such as ascites or obesity. Moreover, both the sensitivity and specificity of ultrasound may be compromised in the setting of slow or irregular blood flow in patients with cirrhosis, which can lead to missed or delayed diagnosis [5]. The reliability of ultrasound in detecting PVT depends not only on the expertise of the individual performing the sonograph but also on the extent of the PVT [6]. In cases where ultrasound is inconclusive or technically difficult, computed tomography (CT) or magnetic resonance imaging (MRI) can be beneficial. Evaluation for hypercoagulable states with laboratory testing should be considered in all patients with PVT and is typically warranted in those without cirrhosis.

Management of PVT with therapeutic anticoagulation aims to achieve recanalization of the portal venous system by preventing thrombus progression, achieving thrombus degradation, and preventing the complications of increased portal venous system pressures [17]. Recanalization can be reasonably achieved in patients with acute PVT, whereas those with chronic PVT are assessed on an individual basis. Significant risks are associated with anticoagulation in the setting of cirrhosis with complications including severe coagulopathies, gastrointestinal bleeding, and intracerebral bleeding; these risks render the use of anticoagulation in this population controversial [17]. Alternative therapeutic options such as catheter-directed thrombolytic therapy have demonstrated efficacy and safety, although it is typically reserved for severe cases and further studies are necessary to delineate patient selection and outcomes [18,19]. Thrombectomy has been attempted with percutaneous mechanical thrombectomy showing better outcomes compared to surgical thrombectomy, although both modalities are not recommended due to high rates of morbidity, mortality, and PVT recurrence [20,21].

Data regarding the safety and efficacy of anticoagulation in patients with cirrhosis is inconclusive, making recommendations for optimal management unclear. Guidelines from the American College of Gastroenterology (ACG) published in 2020 recommend that patients with cirrhosis and acute complete PVT, mesenteric vein thrombosis, or PVT with extension into mesenteric veins should be initiated on therapeutic anticoagulation; either with unfractionated heparin or low-molecular-weight heparin (LMWH) [22]. The anticoagulation agents of choice should be individualized, and clinicians should consider their risk profiles. LMWH is preferred in patients with thrombocytopenia and unfractionated heparin is preferred in patients with decreased kidney function; evidence for these recommendations is low [22]. Anticoagulation with vitamin K antagonists such as warfarin should be avoided in patients with cirrhosis due to its mechanism of action primarily involving the liver. Duration of anticoagulation is typically at least six months with repeat interval imaging to evaluate for resolution of the PVT and recanalization of the portal venous system [3]. Some guidelines, however, recommend three months or indefinite anticoagulation [22]. The benefits of anticoagulation should always be weighed against the risk of bleeding and discussed with the patient to arrive at a management plan through shared decision-making. Furthermore, the ACG guidelines in 2020 recommended that patients with acute PVT who are on therapeutic anticoagulation should also be started on non-selective beta-blocker therapy with propranolol or nadolol for primary prevention of variceal bleeding [22].

A retrospective cohort study conducted by Zhang et al. examined PVT recanalization rates in patients treated with anticoagulation. The study concluded that there was an increased rate of recanalization without an increased rate of bleeding in the patients studied. The authors concluded that therapeutic anticoagulation for acute PVT not only improves the hemodynamics of the portal venous system but also improves long-term liver function. These findings support the efficacy and clinical benefit of therapeutic anticoagulation in patients with cirrhosis and acute PVT [23]. Some experts believe that the available literature is insufficient to determine whether anticoagulation has clinical utility, as a review determined that despite higher recanalization rates and decreased thrombus burden, there was no statistically significant improvement in the mortality rate of patients with cirrhosis and acute PVT who received anticoagulation versus those who did not [3]. However, most experts agree on the consensus that anticoagulation should be initiated in patients with acute decompensated liver disease in whom no etiology besides the acute PVT is attributed to the decompensation.

Chronic PVT is fairly common in patients with cirrhosis; however, its treatment remains unclear with a limited body of evidence surrounding this condition. Patients in whom a chronic PVT is diagnosed have likely developed hemodynamic changes within the portal venous system before the diagnosis, rendering recanalization not useful. Therefore, anticoagulation is not recommended in patients with chronic PVT as it increases the risk of bleeding while not providing clinical benefit [20,24,25]. Decompression shunt surgery offers patients with chronic PVT and subsequent portal hypertension a treatment option to reduce pressures within the portal system and, therefore, reduce the downstream complications; it is also indicated in symptomatic portal hypertensive biliopathy and symptomatic hypersplenism [20,26]. Approaches to the creation of a shunt include TIPS, splenorenal shunt, and mesenteric left portal shunt (also termed a Rex shunt) [27-29]. Liver transplantation is indicated in patients in whom acute decompensation is attributed solely to the development of a PVT without other identifiable reversible factors. However, the presence of PVT is a major obstacle to liver transplantation as it increased surgical complications and confers poor post-surgical outcomes [30].

Bleeding risk is difficult to reasonably predict in patients with cirrhosis, as it is dependent not only on hemostatic abnormalities but also on factors such as the severity of portal pressure and the presence of endothelial dysfunction [17]. If chosen as therapeutic management, anticoagulation for PVT in patients with cirrhosis requires careful monitoring and an individualized approach for each patient’s respective risk to minimize complications [17]. Major contraindications to anticoagulation in these patients include a recent history of bleeding, high-risk gastroesophageal varices, and severe thrombocytopenia [4]. The cutoff for severe thrombocytopenia is controversial, although a consensus lies at a value of <50,000 K/µL [22]. Caution should be exerted in anticoagulating patients with advanced cirrhosis, defined as those with Child-Pugh Class C cirrhosis [4].

## Conclusions

Patients with cirrhosis are at risk for both prothrombotic and antithrombotic complications. Superimposed infections with hepatitis viruses and abnormal flow through the hepatic portal system can vastly increase the risk for PVT. As the diagnosis of acute PVT in patients with cirrhosis can be challenging, clinicians should be keenly aware of the diagnostic limitations with ultrasonography and consider alternate imaging modalities or repeat imaging if clinical suspicion remains high.

Anticoagulation therapy should be considered for patients with cirrhosis on an individual basis for both the prevention and treatment of PVT based on the presence and severity of risk factors. Prompt diagnosis, early intervention, and close monitoring of patients with PVT are crucial for improving clinical outcomes.
